# Effect of cardiac function in patients with gastrointestinal cancer with or without acute kidney injury assessed using a non-invasive impedance cardiography: a case-control study

**DOI:** 10.1186/s12872-023-03533-z

**Published:** 2023-10-04

**Authors:** Huihui Chen, Guolei Zhang, Lei He, Wei Zhou, Shenglei Zhang, Zhe zhe Niu, Jingjing Jin, Mei juan Cheng, Liping Guo, Xiang nan Liang, Rong fang Zhu, Huiran Zhang, Yaling Bai, Jin sheng Xu

**Affiliations:** 1 The Fourth Hospital of Hebei Medical University, Department of Nephrology, Hebei Key Laboratory of Vascular Calcification in Kidney Disease, Hebei Clinical Research Center for Chronic Kidney Disease, 12 Jian kang Road, Shijiazhuang, 050011 P.R. China; 2 Department of Orthopedics, The Third Hospital of Hebei Medical University, Shijiazhuang, P.R. China

**Keywords:** Cardiac function, Acute kidney injury, Gastrointestinal cancer, Non-invasive impedance cardiography, Case-control study

## Abstract

**Objectives:**

This study aimed to analyze the possible causes of changes in cardiac function and investigate the feasibility of clinical assessment of gastrointestinal cancer in patients with or without acute kidney injury (AKI) assessed using a non-invasive impedance cardiography (ICG, Bioz. Cardio Dynamics, USA) to identify independent risk factors.

**Methods:**

Patients admitted to the Fourth Hospital of Hebei Medical University, China, between May 1, 2019, and February 15, 2022, were included in this study. A total of 51 patients with gastrointestinal cancer (31 men and 20 women, mean age 61.1 ± 10.9 years) with or without AKI were evaluated for ICG. A total of 19 patients underwent ultrasound cardiography (UCG) and ICG evaluations.

**Result:**

There was a significant positive correlation between cardiac output (CO), cardiac index (CI), stroke volume (SV), left cardiac work index (LCWI), and ejection fraction (EF) measured using UCG and ICG. The relationship was observed between CO_ICG_ and CO_UCG_ (r = 0.707, P = 0.001), CI_ICG_ and CI_UCG_ (r = 0.718, P = 0.001), SV_ICG_ and SV_UCG_ (r = 0.837, P < 0.001), and LCWI_ICG_ and EF_UCG_ (r = 0.540, P = 0.017). Cardiac function parameters measured using ICG were statistically different between patients with gastrointestinal cancer with or without AKI (P ≤ 0.05). Multivariate analysis revealed that AKI independently affects cardiac function in patients with gastrointestinal cancer.

**Conclusions:**

UCG and ICG methods are significantly associated with cardiac function in patients with or without AKI, and patients with gastrointestinal cancer with AKI are worse than those without AKI. AKI is an independent risk factor for cardiac function in patients with gastrointestinal cancer.

## Introduction

Acute kidney injury (AKI) affects up to half of cancer patients. AKI in cancer patients is a major consequence linked to significantly longer hospital stays and higher expenses for various illnesses [1–4]. The development of AKI in cancer patients continues to be associated with high mortality rates, although survival rates have increased over the last decade [4–8]. A side effect of cancer treatment or the disease itself is the reason for the rising mortality rate [9–11]. AKI is also essential to this process.

As we all know, renal aggravation and heart failure frequently coexist in patients and worsen one another. The term “cardiorenal syndrome” (CRS) was originally used in 2007 by renowned Italian professor Ronco. According to him, type 3 CRS manifests as a sudden decline in renal function that results in acute cardiac dysfunction [12]. Additionally, most research indicates that AKI frequently coexists with multiple organ dysfunction in critically ill cancer patients [7, 8, 12]. However, few studies have examined the cardiac function of patients with gastrointestinal cancer and AKI.

It is difficult to determine the volume status of patients by evaluating their symptoms and performing a physical examination alone. For this reason, impedance cardiography (ICG) was developed as a non-invasive technique that could produce a variety of cardiocirculatory parameters [13–18]. Careful data analysis can identify occult hypovolemia and titrate the optimal dose. Recently, ICG has been suggested as a noninvasive, continuous, operator-independent, and cost-effective approach used in ICUs [19–24]. However, very few studies specifically address the use of ICG in nephrology, especially in patients with gastrointestinal cancer and AKI. The present study aimed to evaluate the two methods—ICG and ultrasound cardiography (UCG)—for assessing cardiac function in patients with gastrointestinal cancer and to identify the factors that may differ between patients with or without AKI.

## Materials and methods

### Design and subjects

This case-control study began on May 1, 2021, and ended on February 15, 2023. During this time, patients with malignant tumors of the gastrointestinal system or AKI were selected from the departments of nephrology, gastroenterology, and hepatobiliary surgery of the Fourth Hospital of Hebei Medical University. This study made use of a single-center database. All patients receiving ultrasound and impedance cardiography had their demographic, laboratory, and clinical data collected by trained physicians.

### Selection of participants and description of data

Patients in our study were divided into case and control groups. ***The case group***: included 25 patients (16 males and nine females) with gastrointestinal cancer and AKI. Inclusion criteria: (a) the primary diseases were cancer of the gastrointestinal system; (b) either the serum creatinine level or the urine output criterion was used to diagnose AKI and, based on the acute kidney injury network (AKIN) stage, determined for each patient; (c) the body mass index (BMI) ranged from 18.5 to 23.9 kg/m^2^, (d) there was no hydrothorax, peritoneal effusion, metal support, or pacemaker in vivo; and (e) volunteering in this study. Exclusion criteria: (a) basal cardiac diseases, including coronary artery disease, pulmonary heart disease, myocardial infarction, and heart failure; (b) BMI < 18.5 kg/m^2^ or > 23.9 kg/m^2^; (c) a history of thoracic operation; (d) severe hypertension mean arterial pressure > 130 mmHg; (e) sepsis shock; (f) chronic kidney disease; and (g) patients who could not cooperate. ***The control group***: included 26 patients (15 males and 11 females) with gastrointestinal cancer without AKI. The case group (c-e) served as the inclusion criteria, and the case group (a-g) served as the exclusion criteria. AKI was categorized at the time of hospitalization using AKIN criteria. A malignancy must be a pathologically proven diagnosis in cancer patients. For this study, only cancer patients with gastrointestinal cancer were considered.

After receiving consent, 19 patients with gastrointestinal cancer underwent UCG (GE Vivid7; Netherlands) and ICG monitoring (Cardio Dynamics, San Diego, California, USA) measurements. The parameters were saved in the electronic memory of the monitor for subsequent analysis. Patients were cared for throughout the study based on the judgment of their regular physicians, who were unaware of the results of the ICG test.

### Measures

After admission, general clinical data such as age, sex, BMI, history of hypertension or diabetes mellites, TNM stage, stage of AKI, etiology of AKI, hemoglobin, leukocyte, platelet, blood albumin, serum creatinine and urea nitrogen were collected. A total of 19 patients (8 cases in the case group and 11 cases in the control group) with gastrointestinal malignant tumors were examined by echocardiography using GE vivid 7-color Doppler ultrasound, and noninvasive hemodynamic monitoring was then performed. Only the cardiac function indicators were recorded for the remaining patients, who were only continually monitored for 5–10 min using the BioZ.com non-invasive hemodynamic monitor produced by Cardio Dynamics in the United States. Cardiovascular parameters such as cardiac output (CO), cardiac index (CI), stroke index (SI), stroke volume (SV), acceleration index (ACI), velocity index (VI), left cardiac work index (LCWI), left cardiac work (LCW), thoracic fluid content (TFC), systemic vascular resistance (SVR), and systemic vascular resistance index (SVRI) were also included.

### Statistical analysis

The Statistical Package for Social Sciences package (version 25.0; SPSS, Chicago, IL) was used to perform the statistical analyses. Noninvasive hemodynamic monitoring and echocardiography were performed to assess cardiac function, and linear correlation analysis was used to assess the corresponding indexes. The corresponding clinical and cardiac function indicators of the collected case group and the control group were subjected to a univariate analysis. Since the measurement data followed a normal distribution (mean ± standard deviation), the median (interquartile distance) can represent a non-normal distribution. For ordered and categorical variables, a nonparametric test was used. To find risk factors affecting cardiac function in patients with gastrointestinal malignancies complicated with AKI, multivariate linear regression analysis was performed on variables with statistical significance (P ≤ 0.05).

## Results

### Characterization of the study population

Over the course of the study, 51 patients were included, with a mean age of 61.00 ± 10.96 years and a confirmed diagnosis of malignancy in each of them. From these patients, (n = 17, 33%) had gastric cancer, (n = 15, 29%) had liver cancer, (n = 7, 14%) had cholangiocarcinoma, (n = 5, 10%) had rectal cancer, (n = 4, 8%) had colon cancer, (n = 3, 6%) had pancreatic cancer. Table 1 shows the basic characteristics of the study population. Table 2 shows cardiac function parameters measured by ICG.

**Table 1:** The basic characteristics of patients with gastrointestinal cancer with or without AKI ^a^

Variables	Cancer with AKI (n = 25)	Cancer without AKI (n = 26)	P^b^
**Clinical**			
Age (years)	61.24 ± 10.95	60.77 ± 10.96	0.88
Male gender, n (%)	16(64%)	15(57.7%)	0.65
Body mass index (kg/m^2^)	19.49 ± 1.78	21.51 ± 1.59	0.40
Hypertension, n (%)	8(32%)	9(34.6%)	0.84
Diabetes mellites, n (%)	4(16%)	5(19.2%)	0.76
Heart rate (beats/min)	84.92 ± 13.41	81.14 ± 13.58	0.31
Systolic blood pressure (mmHg)	137.32 ± 14.45	129.96 ± 11.87	0.26
Diastolic blood pressure (mmHg)	80.96 ± 8.32	77.58 ± 7.21	0.78
Mean arterial pressure (mmHg)	96.56 ± 14.3	91.31 ± 12.5	0.17
TNM stage			
I-II, n (%)	6(24%)	8(30.8%)	0.58
III-IV, n (%)	19(76%)	18(69.2%)	0.59
Stage of AKI			
AKIN1, n (%)	0(0%)	0(0%)	
AKIN2, n (%)	2(8%)	10(38.5%)	<0.05
AKIN3, n (%)	23(92%)	16(61.5%)	<0.05
Etiology of AKI			
Prerenal, n (%)	1(4%)		
Intrarenal, n (%)	1(4%)		
Postrenal, n (%)	8(32%)		
Combined, n (%)	15(60%)		
Laboratory test			
WBC(*10^9^/L)	7.22 ± 0.24	6.75 ± 0.35	0.39
Hgb(g/L)	98(15.1)	105.8(7.1)	0.08
PLT(*10^9^/L)	120.5(29.2)	112.9(32.6)	0.43
Blood albumin (g/L)	25.68 ± 6.09	34.45 ± 7.44	<0.01
Creatinine(μmol/L)	645.5 ± 73.8	69.3 ± 2.41	<0.01
Blood urea nitrogen (mmol/L)	23.4 ± 2.42	4.36 ± 0.35	<0.01

^a^ Categorical data were represented by use numbers (%). Continuous variables conforming to normal distribution were expressed as mean ± standard (x ± s)

The median (P25–P75) was used to express the skewed distribution of continuous variables

^b^ Comparison between patients with gastrointestinal cancer with or without AKI

**Table 2:** Cardiac function parameters measured by ICG^**a**^

Variables	Cancer with AKI (n = 25)	Cancer without AKI (n = 26)	P^b^
**Impedance cardiography**			
Cardiac output (L/min)	4.20 ± 1.29	5.06 ± 1.11	<0.01
Cardiac index (L/min/m^2^)	2.45 ± 0.61	2.97 ± 0.54	<0.01
Stroke volume (ml)	45.8(5.82)	62.78(10.1)	<0.01
Systemic vascular resistance (dyne.s.cm^− 5^)	1761.32 ± 301.66	1374.50 ± 222.32	<0.05
Systemic vascular resistance index (dyne.s/cm^5^/m^2^)	3043.8 ± 598.83	2314.35 ± 277.29	<0.01
Stroke Index (ml/m^2^)	27.08 ± 7.95	37.08 ± 7.50	<0.01
Acceleration Index (/100 s^2^)	64.32(4.02)	70.53(7.28)	<0.05
Velocity index (/1000s)	29.16(4.22)	43.58(10.14)	<0.01
Thoracic fluid content (kOhm^− 1^)	53.45 ± 8.49	33.24 ± 4.46	<0.01
Left cardiac work index (kg/m)	3.02 ± 0.92	3.49 ± 0.90	<0.05
Left cardiac work (kg/m)	5.20 ± 0.76	5.96 ± 0.70	<0.05

^a^ Continuous variables conforming to normal distribution were expressed as mean ± standard (x ± s)

The median (P25–P75) was used to express the skewed distribution of continuous variables

^b^ Comparison between patients with gastrointestinal cancer with or without AKI

Table 3 showed that patients with gastrointestinal cancer and AKI had higher cardiac afterload parameters TFC and SVRI levels than those without AKI, which was statistically significant (*P* < 0.01). In patients with gastrointestinal cancer and AKI, CO, CI, SI, SV, VI, ACI, and LCWI were lower than those without AKI.

**Table 3:** Multiple regression analysis of variables affected the cardiac function

Risk factors	Variable	Unadjusted Coefficients		Standardized Coefficients	P-value
		β	Std.Error	Beta	
SI	Cancer with or without	20.214	4.001	0.585	< 0.01*
TFC	Cancer with or without	20.214	4.001	0.585	< 0.01*

Note: Cancer without AKI was used as a reference

*p<0.01 vs. the control group, statistically significant

Abbreviations: *SI* Stroke index, *TFC* Thoracic fluid content, *AKI* Acute kidney disease

### Comparison between ICG and UCG methods

**Fig. 1:**
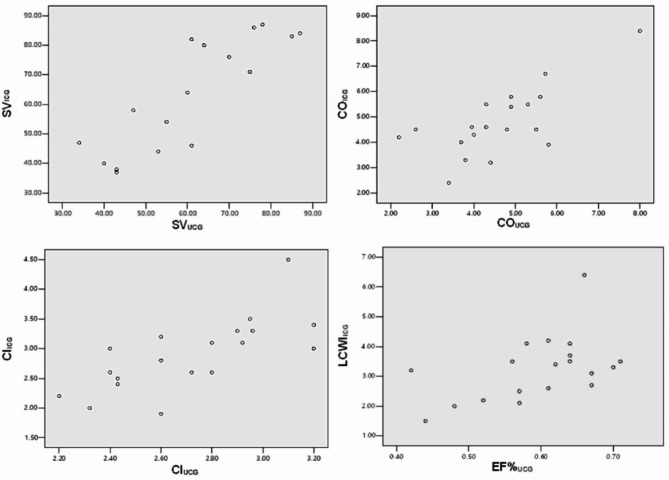
Correlation with SV, CO, CI and LCWI measured by ICG and UCG methods Abbreviations: *SV* Stroke volume, *CO* Cardiac output, *CI* Cardiac index, *LCWI* Left cardiac work index, *ICG* Impedance cardiography, *UCG* Ultrasound cardiography

We examined 19 patients with gastrointestinal cancer (11 men and 8 women), whose average age was 61.8 ± 12.2 years and whose BMI was 21.15 ± 1.20 kg/m^2^. SV_ICG_ ranged from 37 to 86 ml, and SV_UCG_ ranged from 34 to 87 ml. We discovered a significant positive correlation between SV_ICG_ and SV_UCG_ (r = 0.837; *P <* 0.001). CO_ICG_ ranged from 3.2 to 6.5 L.min^− 1,^ and CO_UCG_ ranged from 3.3 to 5.9 L.min^− 1^. There was significant positive significance between CO_ICG_ and CO_UCG_ (r = 0.707; P = 0.001). CI_ICG_ ranged from 2.3 to 3.5 L.min^− 1^.m^− 2^. Additionally, we observed a significant positive correlation between CI_ICG_ and CI_UCG_ (r = 0.718; P = 0.001). EF%_UCG_ ranged from 0.41 to 0.77, and LCWI_ICG_ ranged from 1.4 to 5.1 kg. m. There was a significant positive correlation between CI_ICG_ and EF%_UCG_ (r = 0.519; P = 0.009, LCWI_ICG_, and EF%_UCG_ (r = 0.540; P = 0.017) (Fig. 1).

**Fig. 2:**
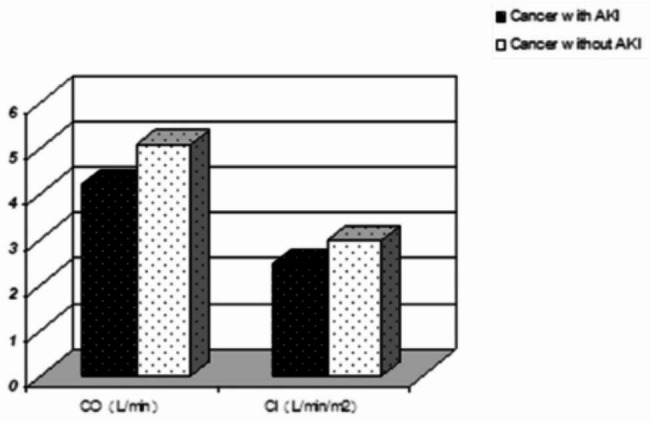
The Cardiac function parameters: CO, CI Measured by Noninvasive hemodynamic monitoring Note: *p<0.05 vs. the control group, statistically significant Abbreviations: *CO* Cardiac output, *CI* Cardiac index

**Fig. 3:**
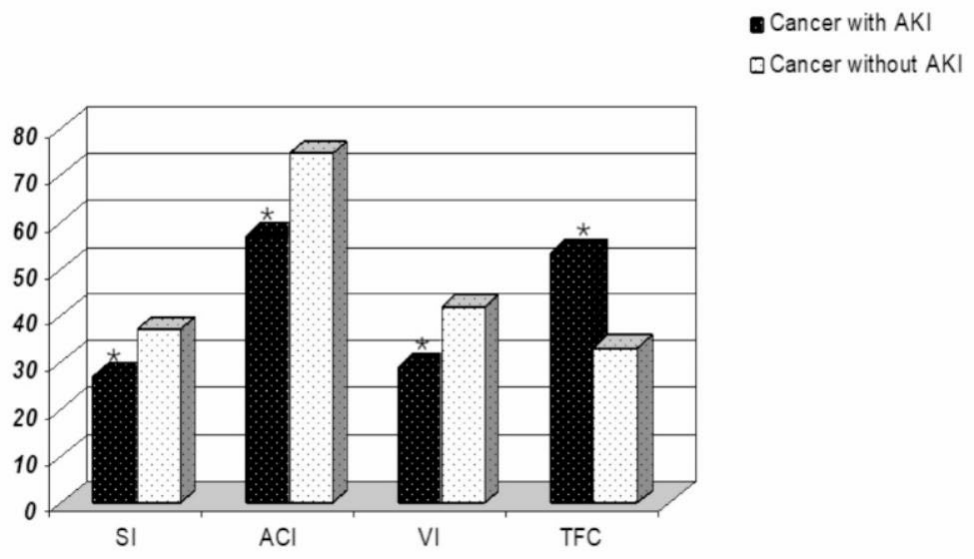
The Cardiac function parameters: SI, ACI, VI, TFC Measured by Noninvasive hemodynamic monitoring Note: *p<0.05 vs. the control group, statistically significant Abbreviations: *SI* Stroke Index, *ACI* Acceleration Index, *VI* Velocity index, *TFC* Thoracic fluid content

**Fig. 4:**
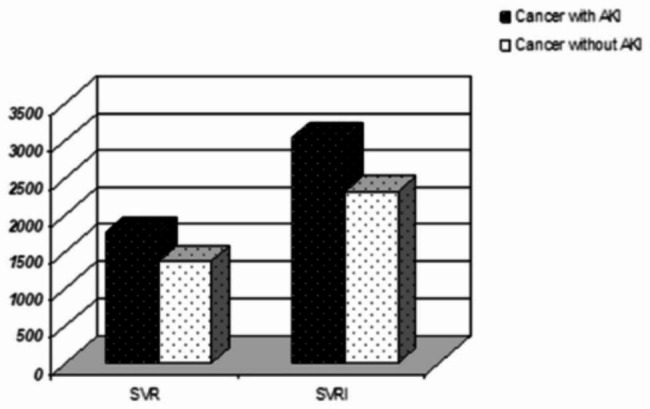
The Cardiac function parameters: SVR, SVRI Measured by Noninvasive hemodynamic monitoring Note: *p<0.05 vs. the control group, statistically significant Abbreviations: *SVR* Systemic vascular resistance, *SVRI* Systemic vascular resistance index

Noninvasive hemodynamic monitoring was used to compare the cardiac function indexes between the case group and the control group: (1) CO (4.20 ± 1.29 vs. 5.06 ± 1.11, P < 0.01); CI (2.45 ± 0.61 vs. 2.97 ± 0.54, P < 0.01) (Fig. 2); SI (27.08 ± 7.95 vs. 37.08 ± 7.50, P < 0.01); SV (45.8(5.82) vs. 62.78(10.1), P< 0.01; ACI (64.32(4.02) vs. 70.53(7.28), P < 0.05) VI (29.16(4.22) vs. 43.58(10.14), P < 0.01; LCWI (3.02 ± 0.92 vs. 3.49 ± 0.90, P < 0.05); and LCW (5.20 ± 0.76 vs.5.96 ± 0.70, P < 0.05) (Fig. 3). According to the findings mentioned above, there was a significant difference between the myocardial contractility, with the case group being significantly lower than the control group. (2) TFC (53.45 ± 8.49 vs. 33.24 ± 4.46, P < 0.01) (Fig. 3), the result demonstrated that the cardiac preload of the case group was significantly higher than that of the control group, and the difference between the two groups was significant. (3) The results revealed that the cardiac afterload of the case group was significantly higher than that of the control group and that the difference between the two groups was statistically significant SVR (1761.32 ± 301.66 vs.1374.5 ± 222.32, P < 0.05); SVRI (3043.8 ± 598.83 vs. 2314.35 ± 277.29, P < 0.01) (Fig. 4). The results revealed that the cardiac afterload of the case group was significantly higher than that of the control group and that the difference between the two groups was statistically significant.

Multivariate regression analysis identified AKI as an independent risk factor for cardiac dysfunction in patients with gastrointestinal cancer after significant factors selected by univariate regression analysis were entered, using SI and TFC as the primary cardiac function index. (r = 0.724, r = 0.585, r = 0.607, *P <* 0.05) (Table 3).

## Discussion

AKI is a severe cancer consequence, significantly increasing morbidity and mortality [1–5, 27–31]. More than half of cancer patients had AKI, which had a dismal prognosis and a mortality rate as high as 78%, according to Maccariello [32] et al. According to a multicenter study [33], patients with severe AKI in the ICU had an in-hospital mortality rate of 60.3%, which suggests that patients with cancer who also have AKI have a higher mortality rate. Malignant tumors alone are not the only factor contributing to the increased mortality rate associated with complicated (or combined) cases of AKI and malignant tumors; AKI also has a significant impact. As can be seen, AKI rarely manifests as an independent disease; rather, it frequently occurs in conjugation with or as a result of the dysfunction of other organs. Hemodynamic instability, significant catabolic and volume overload, a poor prognosis, and a high mortality rate characterize it. Simultaneously, research [34, 35] has revealed that early and timely hemodynamic monitoring of critically ill patients is important to enhance cardiac output and increase kidney blood and urine volume. Given the above, it is crucial for clinical guidance to study the cardiac function status of patients with gastrointestinal malignant tumors complicated by AKI, screen for risk factors affecting the changes in cardiac function, investigate potential mechanisms and clinical significance, and intervene in the early stages of the clinical process to improve patient prognosis and lower mortality and treatment costs. Studies on the cardiac function of patients who have AKI complicated (or combined) by a malignant tumor have not yet been reported.

The current investigation verified that, while evaluating cardiac performance in patients with gastrointestinal cancer, both without AKI, the ICG and the UCG methods showed a statistically significant positive correlation. In 2004, Parrott et al. [25] found a strong correlation (r = 0.85) between changes in EF in patients with heart failure and changes in ICG heart index and systolic time ratio. ICG and CMR have comparable accuracy and concordance rates, as demonstrated by Erifyli et al. [26]; however, ICG cannot be substituted for CMR in patients with breast cancer. Additionally, there was a significant correlation between SV_ICG_, CO_ICG_, SV_UCG,_ and CO_UCG_. Therefore, we conclude that ICG may be a practical, reliable, and economical method for monitoring cardiac function in patients with gastrointestinal cancer. According to most studies, AKI typically develops in the context of multiple organ dysfunction in critically ill cancer patients. This study found that the cardiac function parameters of CO, CI, SV, and ACI measured by ICG in gastrointestinal cancer patients with AKI were lower than those without AKI. In contrast, the TFC, SVR, and SVRI were significantly higher. This was done by comparing the cardiac function parameters in the case and control groups. In other words, heart insufficiency can be brought on by acute renal aggravation in patients with gastrointestinal cancer. At the same time, this study compared the hemoglobin, leukocyte, platelets, diabetes and hypertension history, heart rate and TNM stage of patients in the case group and the control group, and found no difference between the two groups. In addition, our study found differences in blood albumin between the two groups, which may be related to the late TNM stage of the patients, the higher proportion of AKIN stage 3, and the overall severity of the patients’ disease.

What is the mechanism of cardiac dysfunction in cancer patients with AKI? Over the last decade, growing data has revealed that fluid overload is linked to worse outcomes. Additionally, fluid overload is practically given in critically ill patients, particularly in the presence of AKI, and it has been demonstrated that the severity of fluid overload is correlated with a worse clinical outcome [7, 8, 36]. However, only a few studies have shown that cancer patients can experience AKI. In the present study, cardiac function parameters were examined between patients with gastrointestinal cancer with or without AKI, and it was found that TFC was higher in cancer patients with AKI than those without AKI. According to Yerram et al. [8], maintaining the proper fluid balance is essential for managing AKI. We performed multivariate analysis to find the independent determinants of cardiac dysfunction in patients with gastrointestinal cancer. Early ICG monitoring of cardiac function variables may be useful in reducing therapeutic inertia and patient non-adherence to treatment.

### Limitations

This study has some limitations. First, there is insufficient data in this single-center case-control study because there was a small sample size (n = 51). Patients with gastrointestinal malignant tumors complicated (or combined) with AKI have a cardiac function influenced by several clinical factors, requiring a large-sample, multicenter study. Secondly, to minimize the impact of different stages of AKIN on the cardiac function of malignant tumors, the case group selected in this study mainly consisted of patients with gastrointestinal malignancies complicated with AKIN stage 3, and the cause of AKI was mainly postrenal and combined factors. Therefore, the number of patients with AKIN stage 1 and stage 2 and patients with prerenal and intrarenal factors of AKI needs to be increased to more thoroughly prove the influence of different AKIN stages and etiology of AKI on the cardiac function of malignant tumors. Third, the mortality and renal prognostic indexes of hospitalized patients with gastrointestinal malignancies complicated by AKI were not assessed due to the small sample size of this study.

## Conclusion

Non-invasive hemodynamic monitoring and echocardiography correlate well in assessing cardiac function in gastrointestinal malignancies. AKI is closely related to cardiac function in patients with malignant tumors of the gastrointestinal system. AKI is an important risk factor affecting cardiac function changes in patients with malignant tumors of the gastrointestinal system. TFC is significantly increased when AKI occurs in patients with gastrointestinal system malignancies, and it has been hypothesized that internal volume overload may cause decreased cardiac function. Early monitoring of TFC can reduce the risk of cardiac dysfunction and provide new diagnostic and treatment strategies.

## Data Availability

All data analyzed during the current study are included in this article. Further inquiries can be directed to the corresponding author.

## Abbreviations

ACI: Acceleration Index
CI: Cardiac index
CO: Cardiac output
LCW: Left cardiac work
LCWI: Left cardiac work index
SI: Stroke Index
SV: Stroke volume
SVR: Systemic vascular resistance
SVRI: Systemic vascular resistance index
TFC: Thoracic fluid content
VI: Velocity index
